# *H19* lncRNA alters DNA methylation genome wide by regulating S-adenosylhomocysteine hydrolase

**DOI:** 10.1038/ncomms10221

**Published:** 2015-12-21

**Authors:** Jichun Zhou, Lihua Yang, Tianyu Zhong, Martin Mueller, Yi Men, Na Zhang, Juanke Xie, Karolyn Giang, Hunter Chung, Xueguang Sun, Lingeng Lu, Gordon G Carmichael, Hugh S Taylor, Yingqun Huang

**Affiliations:** 1 Department of Obstetrics, Gynecology, and Reproductive Sciences, Yale School of Medicine, New Haven, Connecticut 06510, USA; 2 Department of Surgical Oncology, Affiliated Sir Run Run Shaw Hospital, Zhejiang University School of Medicine, Hangzhou, Zhejiang 310016, China; 3 Department of Obstetrics and Gynecology, Tianjin Renmin Hospital, Tianjin 300000, China; 4 Department of Laboratory Medicine, First Affiliated Hospital of Gannan Medical University, Ganzhou, Jiangxi 341000, China; 5 Department of Obstetrics and Gynecology, University Hospital, Bern 3012, Switzerland; 6 Department of Head and Neck Surgery, State Key Laboratory of Oral Diseases, West China Hospital of Stomatology, Sichuan University, Chengdu, Sichuan 610041, China; 7 Department of Genetics and Genome Sciences, University of Connecticut Health Center, Farmington, Connecticut 06030, USA; 8 Reproductive Medical Center, Henan Provincial People's Hospital, Zhengzhou, Henan 450003, China; 9 Zymo Research Corporation, Irvine, California 92614, USA; 10 Department of Chronic Diseases Epidemiology, Yale School of Public Health, Yale University School of Medicine, New Haven, Connecticut 06520, USA

## Abstract

DNA methylation is essential for mammalian development and physiology. Here we report that the developmentally regulated *H19* lncRNA binds to and inhibits S-adenosylhomocysteine hydrolase (SAHH), the only mammalian enzyme capable of hydrolysing S-adenosylhomocysteine (SAH). SAH is a potent feedback inhibitor of S-adenosylmethionine (SAM)-dependent methyltransferases that methylate diverse cellular components, including DNA, RNA, proteins, lipids and neurotransmitters. We show that *H19* knockdown activates SAHH, leading to increased DNMT3B-mediated methylation of an lncRNA-encoding gene *Nctc1* within the *Igf2-H19-Nctc1* locus. Genome-wide methylation profiling reveals methylation changes at numerous gene loci consistent with SAHH modulation by *H19*. Our results uncover an unanticipated regulatory circuit involving broad epigenetic alterations by a single abundantly expressed lncRNA that may underlie gene methylation dynamics of development and diseases and suggest that this mode of regulation may extend to other cellular components.

## Abstract (web summary)

DNA methylation is an important regulatory process and is essential for correct development and physiology. Here the authors show the long non-coding RNA *H19* regulates methylation by binding and inhibiting S-adenosylhomocysteine hydrolase.

## Graphical abstract

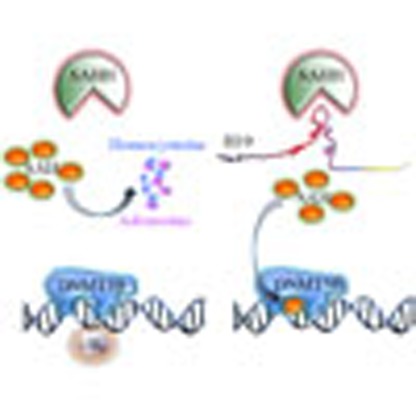

Gene methylation (5-methyl-cytosine), accomplished by the joint action of three S-adenosylmethionine (SAM)-dependent DNA methyltransferases (DNMT1, DNMT3A and DNMT3B), plays a critical role in mammalian development and physiology. Altered gene methylation can cause developmental abnormalities and diseases. Being a dynamic process, methylation at a given gene locus is determined not only by the activity of the DNMTs, which have little sequence specificity, but also by local chromatin structure and modifications (that is, histone methylation and acetylation) that affect their accessibility^1^,^2^,^3^. DNMTs can also be targeted to specific gene loci via direct association with transcription factors^4^. Recent discoveries have marked long noncoding RNAs (lncRNAs) as new important players in DNA methylation regulation. lncRNAs can act in *cis* (at their site of transcription) or in *trans* (diffusing to other loci) to recruit chromatin-modifying complexes such as the Polycomb repressive complex 2 (PRC2) to affect chromatin structure and modifications^5^,^6^. In addition, the noncoding ecCEBPA transcript encoded within the *CEBPA* gene locus binds to DNMT1 and prevents *CEBPA* gene methylation^7^. This methylation blockade by DNMT1-interacting RNAs may extend to other genomic loci, although each of these RNAs acts locally, not genome wide^7^.

SAM-dependent methylation is central to the regulation of numerous biological processes. A wide spectrum of cellular components, including DNA, RNA, lipids, proteins and neurotransmitters, is subjected to methylation by SAM-dependent methyltransferases. SAM serves as the methyl-group donor during transmethylation reactions, yielding S-adenosylhomocysteine (SAH) as a by-product, which is a strong feedback inhibitor of most SAM-dependent transmethylation reactions. In mammals, S-adenosylhomocysteine hydrolase (SAHH) is the only known enzyme that catalyses the hydrolysis of SAH to homocysteine and adenosine, thereby relieving the inhibition^8^. A complete loss of SAHH is embryonic lethal^9^, whereas SAHH dysfunction results in numerous pathological consequences such as developmental abnormalities, neurovascular disorders, myopathy, cancer and childhood death^10^,^11^. Furthermore, inhibition of SAHH elicits antiviral effects^12^. Despite the pivotal roles that SAHH plays in a broad range of biological processes, how its activity is regulated remains poorly understood. Being an extraordinarily conserved enzyme (the human and mouse SAHH proteins share 97% identity), SAHH functions as a tetramer with cofactor NAD^+^/NADH bound to each subunit. Interaction with adenosine or copper inhibits SAHH activity^8^. Recently, lysine acetylation of SAHH has been reported to alter the structure and activity of this enzyme^13^. However, the biological significance of regulation of SAHH by these molecules and by acetylation is unclear. In addition, the possibility of SAHH regulation by other mechanisms remains to be explored.

The developmentally regulated imprinted *H19*, together with its co-regulated gene *Igf2*, plays important roles in embryo development and growth control and has been associated with human genetic disorders^14^. *H19* is highly expressed in human and mouse placentas and fetal tissues as well as in a subset of postnatal and adult tissues including skeletal muscle^15^,^16^, heart^17^,^18^,^19^, haematopoietic stem cells^20^ and endometrium^21^. In addition, aberrant *H19* expression has been detected in diverse human malignancies^22^. *H19* encodes a polyadenylated lncRNA of ∼2.6 kb, which is predominantly cytoplasmic with a minor fraction also found in the nucleus^14^,^23^. *H19* is a multifunctional lncRNA that has activities both in the nucleus and in the cytoplasm. Using genetically modified mouse models and cell culture systems, *H19* has been shown to interact with the methyl-CpG-binding domain protein 1, that recruits repressive histone marks to imprinted network genes to inhibit transcription, thereby contributing to embryo growth regulation^23^. *H19* also serves as a microRNA precursor for miR-675 that acts to inhibit placental growth^24^, maintain adult haematopoietic stem cells^20^, stimulate skeletal muscle differentiation and regeneration^15^ and promote oncogenesis^25^,^26^. The oncogenic property of *H19* is also attributed to its full-length processed transcript that targets PRC2 (through binding to EZH2, the histone lysine methyltransferase component of PRC2) to genes that promote cancer metastasis^27^. In addition to these nuclear functions, *H19* directly binds to the RNA-binding protein K homology-type splicing-regulatory protein in the cytoplasm to control myogenic differentiation^28^. Further, *H19* acts as a molecular sponge for microRNA let-7, contributing to the regulation of muscle differentiation^29^, glucose metabolism^16^, tumour metastasis^30^ and endometrium development^21^.

In this report we show that *H19* binds to SAHH and inhibits its function both *in vivo* and *in vitro*. This interaction prevents SAHH from hydrolysing SAH that blocks DNA methylation by DNMT3B at numerous genomic loci. This differs from what has been described before for other lncRNAs. We propose that this novel epigenetic regulatory mechanism may underlie the DNA methylation dynamics associated with development and diseases and that this mode of regulation may extend to other cellular components.

## Results

### SAHH interacts with *H19* in ribonucleoprotein complexes

**Figure 1:**
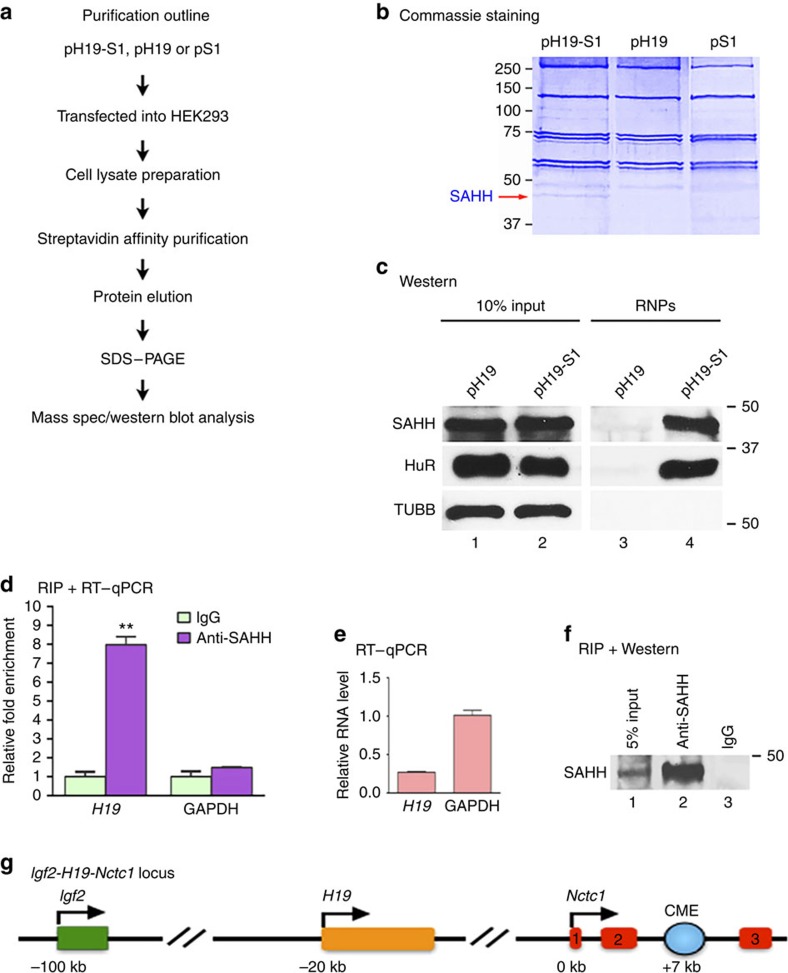
SAHH interacts with H19 in RNPs. (**a**) Schematic outline of purification of *H19*-associated RNPs and protein component identification. (**b**) Image of a Commassie staining gel with protein size markers (in kDa) labelled on the left. The SAHH protein band is marked with a red arrow. (**c**) pH19-S1 or pH19 were transfected into HEK293 cells, followed by affinity purification as outlined in **a**. Purified RNPs were resolved using 10% SDS–PAGE, followed by western blot analysis using rabbit polyclonal antibodies specific for SAHH (top), HuR (middle) or beta-tubulin (bottom). Ten percent input was loaded on lanes 1 and 2. Molecular marker positions in kDa are shown on the right. (**d**) RIP with mouse monoclonal anti-SAHH or pre-immune IgGs from myotube extracts. RNA levels in immunoprecipitates were determined using reverse transcription and qPCR. Levels of *H19* and GAPDH mRNA are presented as fold enrichment in anti-SAHH relative to IgG immunoprecipitates. Student's *t*-tests were used to compare differences between quantitative variables. Numbers are mean±s.d. (*n*=3). **P<0.01. (**e**) Relative RNA levels of *H19* and GAPDH in myotubes. (**f**) Immunoprecipitation using monoclonal anti-SAHH (lane 2) or IgG (lane 3), followed by western blot analysis using a rabbit polyclonal anti-SAHH. Five percent input was loaded in lane 1. The SAHH band is marked. (**g**) Cartoon depiction of the *Igf2-H19-Nctc1* locus (not drawn to scale). The three exons of *Nctc1* are marked as red boxes, and the CME as a blue dot. The transcription start site of *Nctc1* is set as 0 kb.

In search of possible new functions of *H19*, we performed RNA affinity chromatography using an S1 aptamer-tagged *H19* (pH19-S1; untagged pH19 or S1 tag only as negative controls)^29^ to identify *H19*-associated protein components (Fig. 1a). The indicated plasmids were each transfected into HEK293 cells that do not express endogenous *H19*. Purified ribonucleoproteins (RNPs) were resolved using SDS–PAGE, followed by Coomassie blue staining. A protein band with a molecular size of ∼48 kDa that was specific to pH19-S1 (Fig. 1b, left lane marked with a red arrow) was cut out and subjected to protein mass spectrometry analysis. A number of proteins were identified including SAHH, nuclear factor 45, propionyl-CoA carboxylase, methylcrotonoyl-CoA carboxylase, mitochondrial elongation factor Tu (EF-Tu), creatine kinase B-type, 40S ribosomal protein SA, multifunctional protein ADE2 isoform 2 and heterogeneous nuclear RNP D. We decided to focus on SAHH for several reasons. First, SAHH belongs to a large family of proteins that use NAD(P)^+^/NAD(P)H as cofactors via the dinucleotide-binding Rossmann-fold (a protein structural motif that binds nucleotides, especially the cofactor NAD)^8^. Second, a dozen enzymes harbouring the Rossmann-fold have been reported to bind polyadenylated RNAs in HeLa cells^31^. Third, the Rossmann-fold is an RNA-binding domain for both glyceraldehyde-3-phosphate dehydrogenase (GAPDH) and lactate dehydrogenase (LDH) and mediates their binding to AU- and U-rich RNA elements^32^,^33^. Finally, SAHH is the only mammalian enzyme capable of breaking down SAH, a potent product inhibitor of most SAM-dependent transmethylation reactions.

**Figure 2:**
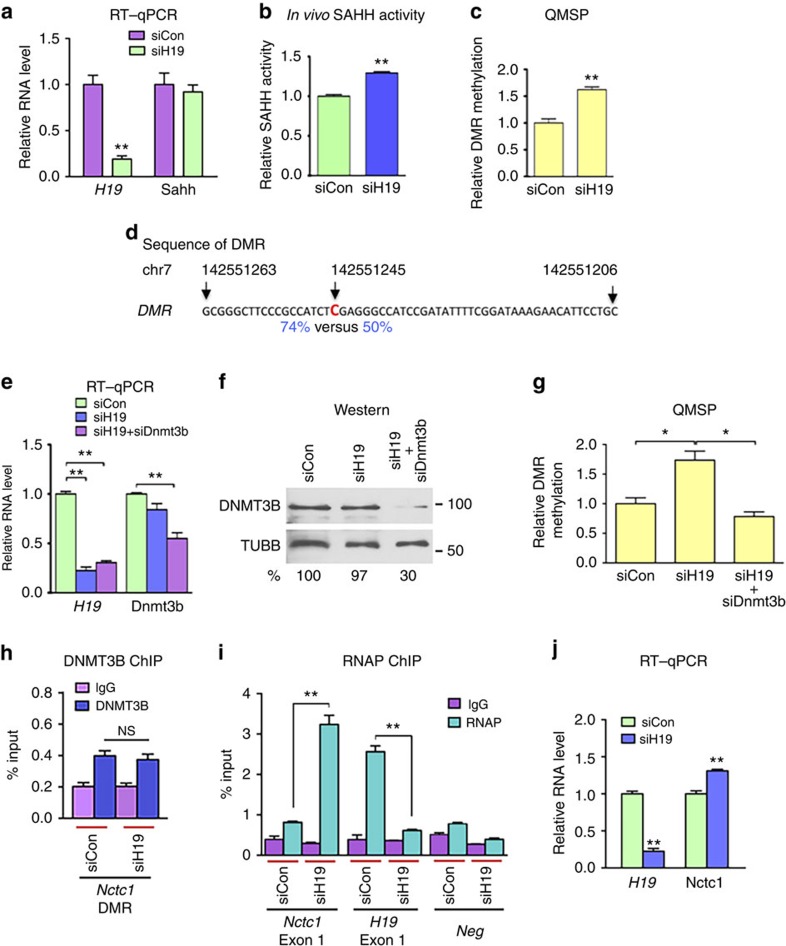
DNMT3B contributes to SAHH-mediated, H19-dependent methylation change in CME. (**a**) Myotubes were transfected with siCon or siH19, followed by RNA extraction and RT–qPCR analysis 48 h later. Relative RNA levels of *H19* and Sahh are presented. (**b**) *In vivo* SAHH activity assessed 48 h post-siRNA transfection, as described in **a**. (**c**) Relative DMR methylation assessed using QMSP. (**d**) Sequence of the DMR amplified using the QMSP primers, with the differentially methylated cytosine highlighted in red. Numbers above the sequence mark positions of the indicated nucleotides in the chromosome. Numbers below indicate percentage of methylation in siH19 (left) versus siCon (right) cells, as determined using genome-wide methylation analysis. (**e**) Myotubes were transfected with siCon, siH19 or siH19 plus siDnmt3b, followed by RNA extraction and RT–qPCR analysis 48 h post transfection. Relative RNA levels of *H19* and Dnmt3b are presented. (**f**) Western blot results representative of three independent transfection experiments (performed as described in **e**) are shown. Antibodies specific for DNMT3B and beta-tubulin were used on the top and bottom blots, respectively. Numbers underneath the blots indicate DNMT3B protein levels after normalization against TUBB loading controls with that in siCon cells arbitrarily set as 100%. (**g**) Myotubes were transfected with siCon, siH19 or siH19 plus siDnmt3b. DMR methylation was assessed 48 h post transfection using QMSP. (**h**) DNMT3B occupation at CME. Myotubes were transfected with siCon or siH19. ChIP analysis was performed 48 h later using anti-DNMT3B antibody to evaluate binding of DNMT3B at the DMR. ns, not statistically different. (**i**) Association of activated RNAP with *Nctc1* and *H19*. Myotubes were transfected as described in **h**. ChIP analysis was performed using anti-RNAP antibody. (**j**) Relative RNA levels of H19 and Nctc1 following siRNA transfection. Student's *t*-tests were performed to compare differences between quantitative variables. Numbers are mean±s.d. (*n*=3). *P<0.05, **P<0.01.

Western blot analysis of affinity-purified RNPs revealed association of SAHH with pH19-S1 but not with pH19 (Fig. 1c, top blot, compare lane 3 with lane 4). The RNA-binding protein HuR, previously shown to bind *H19* (ref. 24), was also in complex with pH19-S1 under the same conditions (middle blot, lane 4), while beta-tubulin was not (bottom blot, lane 4). Taken together, these results supported the notion that the SAHH–H19 interaction was specific. Next, we performed RNA immunoprecipitation (RIP) experiments using a monoclonal antibody specific to SAHH with myotubes derived from mouse myoblasts to determine whether SAHH associates with endogenous *H19* Both *H19* and SAHH were predominantly localized to the cytoplasm of the myotubes (Supplementary Fig. 1). We observed a nearly eightfold enrichment of H19 in SAHH-containing RNPs relative to control IgG immunoprecipitates (Fig. 1d, left column), whereas GAPDH mRNA, expressed at approximately fourfold higher level than that of *H19* (Fig. 1e), did not detectably associate with the SAHH-containing RNPs (Fig. 2e, right column). The specificity of the monoclonal SAHH antibody was confirmed using RIP and western blot analysis (Fig. 1f). As expected, we observed preferential enrichment of *H19* in HuR-containing RNPs using a polyclonal HuR antibody under the same conditions (Supplementary Fig. 2). Collectively, we concluded that SAHH interacts with H19 in RNPs in both human and mouse cells.

### *H19* knockdown activates SAHH

What could be the functional significance of the SAHH–*H19* interaction? We hypothesized that binding to *H19* might interfere with SAHH's ability to hydrolyse SAH to homocysteine and adenosine. To test this possibility, we used the well-characterized, imprinted *Igf2-H19-Nctc1* gene locus in mouse skeletal muscle cells as a model system. The regulation of expression of these genes requires a shared enhancer that is located 20 kb downstream of *H19* (or 100 kb downstream of *Igf2*)^34^. This enhancer functions in an unique manner in that it needs both a short core element of ∼300 bp (called core muscle enhancer, or CME) and transcription of the lncRNA-encoding gene *Nctc1* within the same enhancer region^35^ (Fig. 1g). The CME resides within the gene body of *Nctc1* in the second intron and is 7 kb downstream of the *Nctc1* promoter. In adult muscle cells where *Nctc1* has lost its imprinting^36^, activation of the *Nctc1* promoter by CME has a negative impact on *H19* expression. Promoter competition (the *Nctc1* promoter recruits RNA polymerase II (RNAP) for its own transcription at the expense of activation of the *H19* promoter) has been proposed as a possible underlying mechanism^36^. Indeed, in myotubes differentiated *in vitro* from mouse myoblasts *Nctc1* expression was inversely correlated with that of *H19*. Consistent with the promoter competition model, association of RNAP with *Nctc1* was also inversely correlated with *H19* transcriptional activation^36^. Intriguingly, the CME contains a 219-bp CpG island with unknown function. We decided to use this well-characterized myotube system, where a negative impact of *Nctc1* promoter activity on *H19* transcription has been demonstrated^36^, to test the hypothesis that the SAHH–*H19* interaction may affect CME methylation, which in turn influences the transcriptional activity of *Nctc1* and *H19*.

Thus, we downregulated *H19* using an *H19*-specific short interfering RNA (siRNA; siH19 (refs 16, 29)) in myotubes differentiated from mouse myoblasts, followed by *in vivo* SAHH activity assessment using homocysteine as a readout^37^. When the *H19* level was reduced to ∼20% of the control (Fig. 2a, left column) without significantly affecting the level of SAHH mRNA (right column), an ∼20% increase in SAHH activity was observed (Fig. 2b). These results supported the view that *H19* association inhibits SAHH's ability to hydrolyse SAH, a strong inhibitor of SAM-dependent methyltransferases including DNMTs. We hypothesized that increased SAHH activity would activate DNMTs, which otherwise would be inhibited by SAH, leading to increased gene methylation. Indeed, we observed a 30% increase in a differentially methylated region (DMR) within the CME as determined by quantitative methylation-specific PCR (QMSP) analysis (Fig. 2c). The PCR primers were designed to cover this DMR based on our single-nucleotide resolution genome-wide DNA methylation analysis (Fig. 2d, and see below). Primers that cover another DMR (DMR-2) of CME showed similar results (Supplementary Fig. 3). Next, we wanted to test whether DNMT3B might be responsible for this increased methylation. It was previously reported that in human skeletal muscle cells the enzymatic activity (but not the protein level) of DNMT3B increased in response to free fatty acids, resulting in promoter hypermethylation of *PGC-1a* (ref. 38). Further, recent genome-wide DNA methylome studies revealed a strong association of DNMT3B with gene body methylation and its positive correlation with gene expression^1^,^39^. Thus, CME methylation was evaluated in the presence of double siRNA knockdown. In the *H19*/*Dnmt3b* double knockdown group, *Dnmt3b* was silenced by ∼40% at the RNA (Fig. 2e, right column) and ∼70% at the protein levels (Fig. 2f, top blot). While *H19* single knockdown expectedly increased CME methylation (Fig. 2g, compare middle bar with left bar) without altering the DNMT3B protein level (Fig. 2f, top blot, compare middle lane with left lane), the double knockdown restored methylation to the control level (Fig. 2g), suggesting that DNMT3B contributed to increased CME methylation induced by *H19* knockdown. A second siRNA (siDnmt3b-2) targeted to a different region of *Dnmt3b* produced similar results (Supplementary Fig. 4), confirming the specificity of the Dnm3b siRNA effects. Further, we carried out chromatin immunoprecipitation (ChIP) analysis using a DNMT3B antibody shown to be highly specific for DNMT3B in a genome-wide ChIP study^1^. Results revealed no significant change in DNMT3B occupancy at the DMR when *H19* was downregulated (Fig. 2h), although a readily detectable change in RNAP association with *Nctc1* was observed under the same conditions (see below). These results suggested that it was the activity and not the chromatin residence of DNMT3B that was responsible for the methylation change. This result is in line with a previous report showing that a change in the enzymatic activity of DNMT3B alone was sufficient to induce hypermethylation of *PGC-1a* in human skeletal muscle cells^38^. Collectively, these results suggested that reducing *H19* level de-represses SAHH and enables it to act on SAH, thereby relieving inhibition of DNMT3B by SAH and allowing DNMT3B to methylate CME.

### CME methylation correlates with enhanced *Nctc1* transcription

To determine the effect of CME methylation on *Nctc1* transcription, we performed ChIP analysis using an anti-Ser-5(P)-RNAP antibody that specifically recognizes activated RNAP as previously described^35^,^36^. We compared binding of activated RNAP to *Nctc1* between control siRNA (siCon)- and siH19-transfected myotubes to assess relative transcription activity of *Nctc1* (refs 35, 36). We observed an approximately fourfold enrichment in RNAP at *Nctc1* in siH19- versus siCon-transfected myotubes (Fig. 2i), suggesting an increase in *Nctc1* transcription that was further supported by the concomitant increase in the Nctc1 RNA level (Fig. 2j). This positive correlation between CME methylation and *Nctc1* transcription was not surprising because increased gene body methylation has been associated with increased transcription^1^,^3^,^39^,^40^. We further noted a significant decrease in RNAP association with *H19* in siH19-transfected myotubes (Fig. 2i, middle column, compare right green bar with left green bar). These results recapitulated the previously reported observation showing an inverse relationship of transcriptional activity between *Nctc1* and *H19* (ref. 36) and suggested a novel functional role of CME methylation in the regulation of *Nctc1* and *H19*.

### SAHH is required for *H19*-dependent CME methylation change

**Figure 3:**
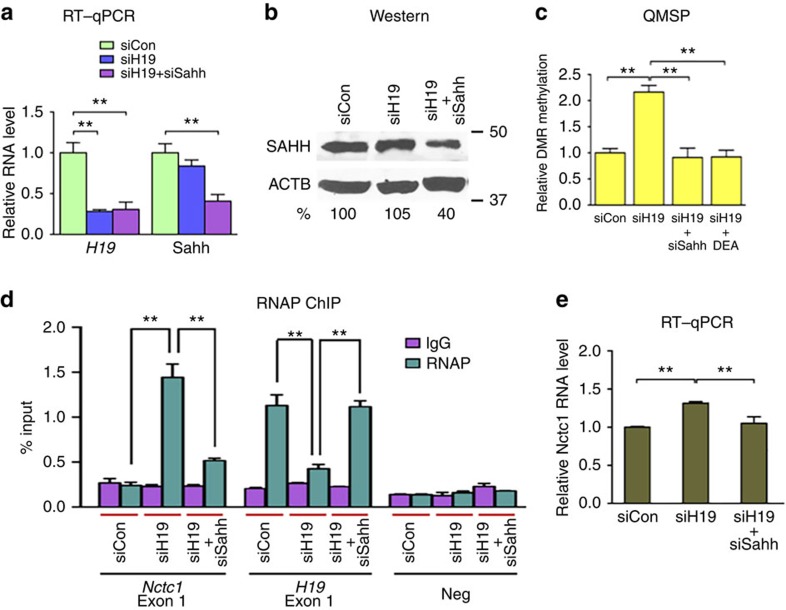
SAHH is required for H19-dependent alteration in CME methylation and *Nctc1* transcription. (**a**) Myotubes were transfected with siCon, siH19 or siH19 plus siSahh, followed by RNA extraction and RT–qPCR analysis 48 h later. Relative RNA levels of *H19* and Sahh are presented. (**b**) Western blot results representative of three independent transfection experiments (performed as described in **a**) are shown. Antibodies specific for SAHH and beta-actin were used on the top and bottom blots, respectively. Numbers indicate SAHH protein levels after normalization against beta-actin loading controls with levels in siCon cells arbitrarily set as 100%. (**c**) Myotubes were transfected with siCon, siH19, siH19 plus siSahh or siH19 plus incubation with 10 μM of DEA 20 h post transfection. DMR methylation was assessed 48 h post transfection using QMSP. (**d**) Association of activated RNAP at *Nctc1* and *H19*. Myotubes were transfected with siCon, siH19 or siH19 plus siSahh. ChIP analysis was performed 48 h post transfection. (**e**) Relative Nctc1 RNA levels measured 48 h post transfection, as described in **d**. Student's *t*-tests were performed to compare differences between quantitative variables. Numbers are mean±s.d. (*n*=3). **P<0.01.

To provide further evidence that increased CME methylation induced by *H19* knockdown was indeed mediated by SAHH, double knockdown experiments using a Sahh-specific siRNA^10^ were carried out. In the *H19*/*Sahh* double knockdown cells, *Sahh* was silenced by ∼60% at both the RNA (Fig. 3a, right column) and protein levels (Fig. 3b, top blot). While *H19* single knockdown increased methylation, *H19*/*Sahh* double knockdown abrogated this effect (Fig. 3c). The abrogation effect was also observed when a pharmacological SAHH inhibitor D-Eritadenine (DEA; Supplementary Fig. 5)^41^,^42^ was used (Fig. 3c). These results further supported the notion that SAHH is required for H19-mediated CME methylation change.

As increased CME methylation following *H19* knockdown correlated with increased *Nctc1* transcription (Fig. 2g–j), we predicted that *H19* and *Sahh* double knockdown would reverse this effect, given that increased CME methylation was dependent on SAHH activation (Fig. 3c). Consistent with our earlier observations (Fig. 2i, left column; and Fig. 2j, right column), *H19* single knockdown increased RNAP association with *Nctc1* (Fig. 3d, left column, compare middle green bar with left green bar) with a concomitant increase in the Nctc1 RNA level (Fig. 3e, compare middle bar with left bar). In the *H19* and *Sahh* double knockdown cells, RNAP association with *Nctc1* was significantly reduced compared with that in the *H19* single knockdown cells (Fig. 3d, left column, compare right green bar with the middle green bar), and there was a decrease in the Nctc1 RNA level as well (Fig. 3e). Moreover, the inverse relationship of RNAP association between *Nctc1* and *H19* was lost in the double knockdown cells (Fig. 3d, compare between the left and middle columns). Collectively, these results strongly suggested that *H19* induced CME methylation change through the action of SAHH, which in turn affected the transcription of *Nctc1* and *H19*.

### U-rich element binding inhibits SAHH activity *in vitro*

**Figure 4:**
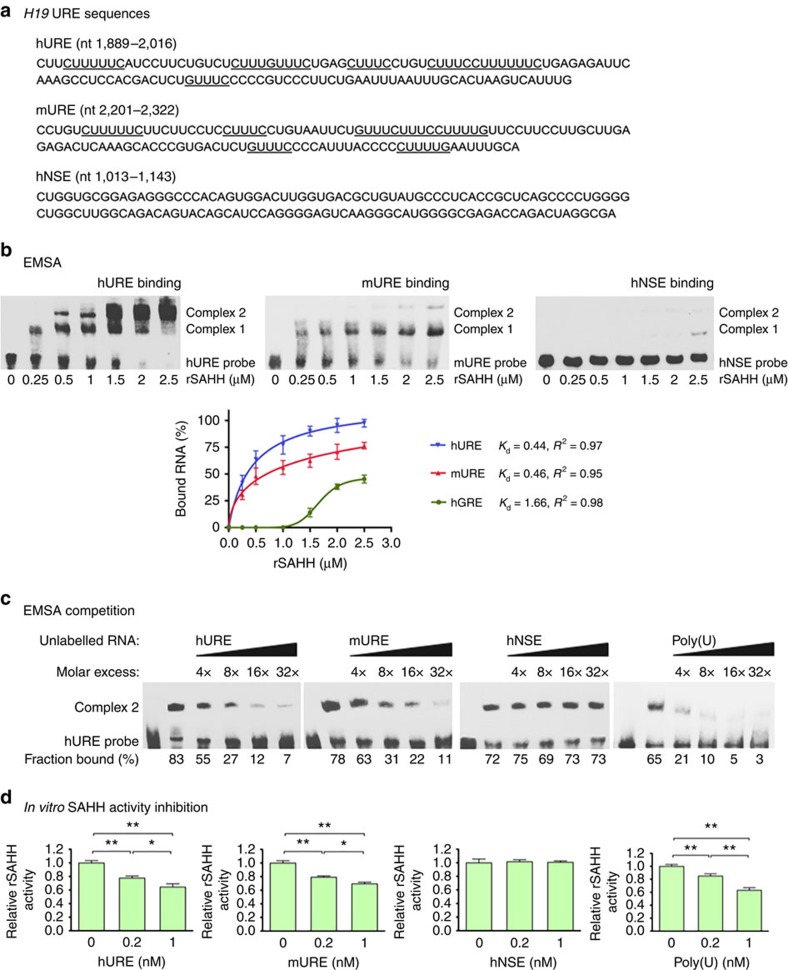
Binding to U-rich elements of *H19* inhibits SAHH activity *in vitro*. (**a**) Sequences of human and mouse *H19* fragments used in EMSA and *in vitro* SAHH activity assays, with the U-rich stretches underlined. Numbers in parentheses mark the nucleotide positions of the sequences relative to the transcription start sites of H19. (**b**) EMSA results using biotin-labelled hURE, mURE or hNSE with increasing concentrations of rSAHH. The positions of unbound RNA and RNA–protein complexes are marked to the right. Band intensities were quantified from three independent experiments and used to calculate the *K*_d_ values displayed in the bottom panel. (**c**) EMSA results with biotin-labelled hURE and 2 mM of rSAHH. The amounts of unlabelled competitors added in relative molar excess are indicated on top. Band intensities were calculated and presented as percentage of fraction bound. Numbers are average of two independent experiments. (**d**) *In vitro* rSAHH activity assay showing dose-dependent inhibition of the hydrolytic activity of rSAHH by the UREs and poly(U). Student's *t*-tests were used to compare differences between quantitative variables. Numbers are mean±s.d. (*n*=4). **P<0.01, *P<0.05.

To further delineate the molecular nature of SAHH–*H19* interaction, we took an *in vitro* approach to map binding sites of SAHH on *H19*. Both GAPDH and LDH bind to AU- and U-rich RNA elements via the Rossmann-fold^32^,^33^. SAHH has a Rossmann-fold that binds cofactor NAD^+^, which is required for maintaining its quaternary structure and catalytic function^8^. We hypothesized that SAHH may bind U-rich elements (URE), as we noticed stretches of UREs flanked by C or G near the 3′-end of both human and mouse *H19* (Fig. 4a). To test this hypothesis, we performed electrophoretic mobility shift assays (EMSAs) using purified recombinant human SAHH protein (rSAHH) and 3′-end biotin-labelled *H19* RNA fragments. At low rSAHH concentrations, only one complex (complex 1) formed with both human (hURE) and mouse (mURE) fragments (Fig. 4b, top two panels on the left). As rSAHH concentration rose, an additional complex (complex 2) appeared, suggesting simultaneous binding of more than one molecule of rSAHH to multiple U-rich sequences. We estimated the *K*_d_ values of rSAHH binding to the human and mouse UREs to be 0.44 and 0.46 μM, respectively (Fig. 4b, bottom panel), similar to the *K*_d_ value of LDH for the granulocyte–macrophage colony-stimulating factor RNA AU-rich element, which was reported to be 0.5 μM (ref. 33). The apparently slightly lower *K*_d_ value of hURE was also reflected by competition experiments showing that unlabelled hURE competed better for binding to rSAHH than the unlabelled mURE (Fig. 4c, left two panels). A non-U-rich fragment (hNSE) randomly chosen from human H19 failed to efficiently compete for rSAHH binding (Fig. 4c, third panel), which was consistent with its poor rSAHH-binding affinity (Fig. 4b). Further, the poly(U) ribohomopolymers exhibited the highest activity in reducing complex formation (Fig. 4c, fourth panel). Together, these results showed that purified rSAHH was capable of direct binding to UREs in both human and mouse *H19* *in vitro*.

As *H19* binds and inhibits SAHH activity *in vivo* (Figs 1c,d and 2b), we tested effects of *H19* fragments on the ability of rSAHH to hydrolyse SAH *in vitro*. Both hURE and mURE inhibited the hydrolytic activity of rSAHH in a dose-dependent manner, whereas hNSE did not (Fig. 4d). This was in line with the observation that both UREs had significantly higher affinity for rSAHH than hNSE (Fig. 4b,c). As expected, poly(U) also efficiently inhibited rSAHH activity (Fig. 4d). Combined with our *in vivo* data (Figs 1c,d and 2b), these results strongly support our hypothesis that SAHH binding to *H19* interferes with its ability to hydrolyse SAH *in vivo*.

### *H19*-dependent SAHH inhibition exerts global effects

As SAHH can potentially affect SAM-dependent methyltransferases other than DNMT3B, one would predict that *H19* knockdown would lead to global DNA methylation changes. Methylation at a particular cytosine residue within a gene locus is influenced both by the activity of DNMTs and by other epigenetic marks that affect their accessibility^1^,^2^,^3^. SAHH activity change could not only directly affect the activity of DNMTs, but also other epigenetic marks, which in turn might indirectly affect gene methylation. Further, not all methyltransferases display an equal sensitivity to SAHH inhibition^8^. Thus, increased SAHH activity as a result of *H19* knockdown is expected to produce three different outcomes: some genomic loci would become hypermethylated; some hypomethylated; and some would not change, relative to control knockdown.

As a proof-of-principle that *H19* modulates gene methylation through SAHH, we performed global DNA methylation studies to identify genes whose methylation might be affected in a way similar to that of *Nctc1*. Mytotubes were transfected with siH19 (or siCon as a control), and DNA was extracted 48 h later and subjected to genome-scale DNA methylation mapping using the platform of an improved version of Reduced Representation Bisulfite Sequencing. This platform offers detection of —three to four million unique CpG sites, allowing >85% coverage of all annotated CpG islands and >80% of all gene promoters for a maximal amount of methylation data. It allows the identification and analysis of DMRs between samples at single-nucleotide resolution. As expected, following *H19* knockdown we observed extensive genome-wide methylation pattern changes relative to siCon cells, with some genes showing increased methylation, others showing decreased methylation and a third group with no significant change (Supplementary Fig. 6). We hypothesized that in the group of genes showing increased methylation, some were likely affected by increased activity of DNMT3B as a direct result of SAHH activation. To test this possibility, we selected five genes showing strong hypermethylation with siH19 (Supplementary Data 1). Since SAHH dysfunction is associated with human diseases^8^,^10^,^11^, we selected genes having important cellular functions or implicated in diseases. *Bmp8b* (bone morphogenetic protein 8b) has been reported to act as a signalling molecule in energy balance regulation in both brown adipocytes and neurons, and *Bmp8b* null mice exhibit decreased rate of metabolism and impaired thermogenesis^43^. *Casp2* (Caspase 2) is a member of the caspase family that mediates cellular apoptosis through the proteolytic cleavage of specific protein substrates^44^. In addition to its well-defined role in cell death, *Casp2* has been implicated in neurodegenerative diseases, tumorigenesis, metabolism and aging^44^. *Cdt1* (chromatin licensing and DNA replication factor 1) is a component of the prereplicative complex that assembles on genomic DNA at origins of replication. Mutations in this gene have been associated with growth failure in primordial dwarfism^45^. *Foxa2* (forkhead box A2) is a DNA-binding transcription factor essential for the development of endoderm-derived organs including the liver and pancreas. *Foxa2* has been implicated in blood glucose homeostasis by controlling the expression of multiple genes in pancreatic beta-cells^46^. *Setd2* (SET domain containing 2) is a member of the SAM-dependent histone lysine methyltransferase family (which also includes EZH2, a component of PRC2) known to be important for chromatin modification^2^,^47^. SETD2 interacts with the Huntington's disease (HD) protein Huntingtin and has been implicated in the pathogenesis of HD, a neurodegenerative disorder characterized by loss of striatal neurons^48^.

**Figure 5:**
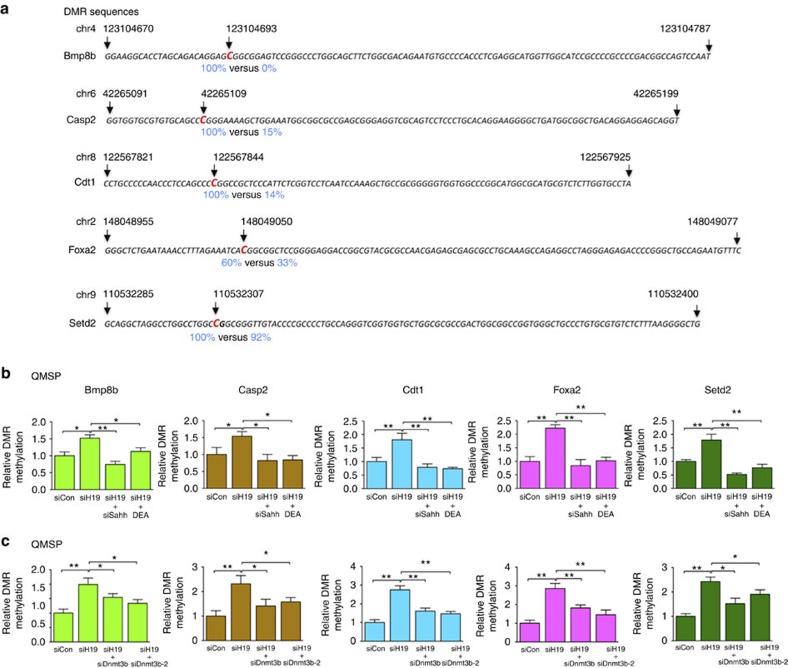
SAHH is required for H19-dependent methylation change in the DMRs of other genes. (**a**) Sequences of the DMRs of the indicated genes amplified using QMSP primers. The differentially methylated cytosine residues are highlighted in red. Numbers above the sequences mark the positions of the indicated nucleotides in the chromosomes. Numbers below (in blue) indicate percentage of methylation in the siH19 (left) versus siCon (right) cells as determined by genome-wide methylation mapping. (**b**) Myotubes were transfected with siCon, siH19, siH19 plus siSahh or siH19 plus incubation with 10 μM of DEA 20 h post transfection. DNAs were isolated 48 h post transfection and analysed using QMSP. (**c**) Myotubes were transfected with siCon, siH19, siH19 plus siDnmt3b or siH19 plus siDnmt3b-2. DNAs were isolated 48 h later and analysed using QMSP. Student's *t*-tests were used to compare differences between quantitative variables. Numbers are mean±s.d. (*n*=3). **P<0.01, *P<0.05.

To determine whether the increased methylation in these genes as revealed by genome-wide analysis was SAHH-dependent, myotubes were transfected with siH19 or siCon, with or without co-transfection of siSahh or treatment of SAHH inhibitor DEA. QMSP analysis was carried out 48 h post transfection using primers that specifically amplify a single DMR of each respective gene (Fig. 5a). While *H19* knockdown increased DMR methylation, its combination with siSahh or DEA rescued methylation to the level of the control (Fig. 5b). These results strongly suggested *H19*-mediated, SAHH-dependent regulation of methylation at these sites, thus validating our genome-wide data. To further address whether DNMT3B might contribute to the increased methylation, *H19*/*Dnmt3b* double knockdown experiments were carried out, followed by QMSP analysis. As shown in Fig. 5c, siH19 in combination with either Dnmt3b siRNA was able to partially or fully restore methylation to the level of the siCon control. The partial rescue by the Dnmt3b siRNAs suggested that other DNMTs may contribute to the methylation of these sites, which warrants future investigation. It is noteworthy that, while a single DMR methylation change may seem small, the combined effects from multiple DMRs in a single gene (Supplementary Table 1 and Gene Expression Omnibus (GEO) accession number GSE73014) could potentially be synergistic, leading to bigger biological effects.

**Figure 6:**
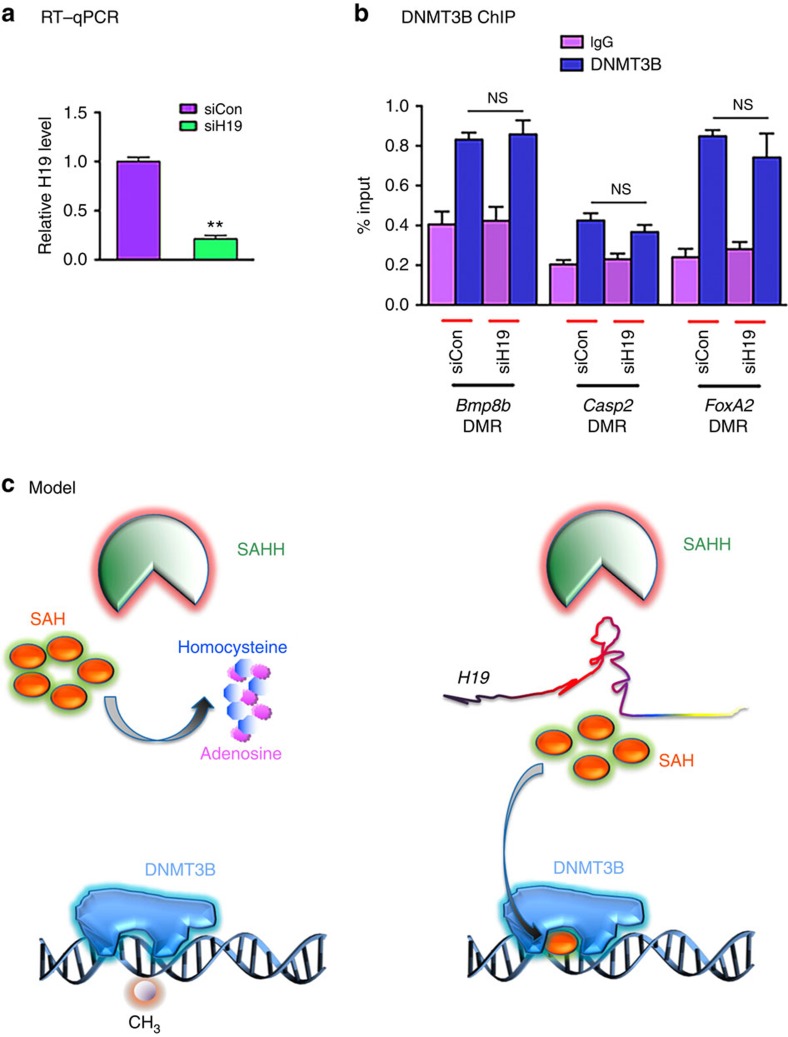
A model for *H19*/SAHH-dependent regulation of gene methylation. (**a**) Myotubes were transfected with siCon or siH19, followed by RNA extraction and RT–qPCR analysis 48 h later. Relative *H19* level is presented. Student's *t*-tests were used to compare differences between quantitative variables. Numbers are mean±s.d. (*n*=3). **P<0.01. (**b**) DNMT3B ChIP at the DMRs of the indicated genes. ns, not statistically different. (**c**) In the absence of H19 (left panel), SAHH hydrolyses SAH to homocysteine and adenosine, and DNMT3B is active in DNA methylation. When *H19* is present (right panel), SAHH activity is attenuated because of its association with H19. This leads to accumulation of SAH, which binds to DNMT3B and prevents it from methylating DNA.

Next, DNMT3B ChIP analysis was conducted following siRNA transfection (Fig. 6a). While no DNMT3B association was detected at the DMRs of *Cdt1* and *Setd2*, for the remaining three genes tested, no change in DNMT3B occupancy at the respective DMRs was observed following *H19* knockdown (Fig. 6b). These results supported the notion that the *H19* knockdown-induced increase in methylation was due to changes in DNMT3B activity rather than its chromatin association.

*H19* regulates gene expression via multiple mechanisms, both in the nucleus and in the cytoplasm. Thus, *H19* knockdown could alter its nuclear interaction with methyl-CpG-binding domain protein 1 and/or EZH2 to affect gene transcription^23^,^27^. In the cytoplasm, *H19* knockdown could change the dynamic association of K homology-type splicing-regulatory protein with its target mRNAs, leading to their stability changes^28^. The mRNA levels of genes targeted by let-7 could also be affected, as *H19* acts as a molecular sponge for let-7 (ref. 29). Finally, *H19* could modulate transcription by affecting gene methylation through interaction with SAHH (this report). Thus, the effects of *H19* knockdown on genome-wide transcript levels would represent a summation of contributions from the different mechanisms outlined above as well as from other yet unknown mechanisms. Consistent with this view, our RNA-sequencing (RNA-seq) analysis revealed profound changes in transcript profiles between siCon- and siH19-transfected myotubes (Supplementary Data 2 and GEO accession number GSE73014). While a number of these are consistent with alterations in DNA methylation profiles, the multiplicity of *H19* functions does not at this time allow definitive mechanistic connections to be established.

## Discussion

This study uncovers a novel regulatory circuit of gene methylation dictated by the interaction between *H19* and SAHH. Using the *Igf2-H19-Nctc1* gene locus in mouse muscle cells as a model, we demonstrate that binding of SAHH to *H19* interferes with its ability to hydrolyse SAH, which is a potent product inhibitor of SAM-dependent methyltransferases. This leads to decreased DNMT3B-mediated methylation within the *Nctc1* gene body and subsequently reduced transcriptional activity of the *Nctc1* promoter. We show that UREs in *H19* that display preferential binding to SAHH also inhibit the catalytic activity of SAHH, suggesting that the basis for this preferential interaction is recognition of the UREs. Further, we demonstrate that this *H19*–SAHH-dependent regulation occurs at other genomic loci, suggesting global effects of this regulation. On the basis of these findings, we propose the model illustrated in Fig. 6c. To our knowledge, this represents the first case in which a single lncRNA acts in *trans* to alter the epigenetic landscape in a genome-wide manner.

Importantly, our findings offer novel insights into DNA methylation regulation associated with development and pathological conditions. Specifically, our results suggest that the enzymatic activity of chromatin-bound DNMT3B might be subjected to modification by *H19* through the *H19*/SAHH pathway. Interestingly, DNMT3B (but not DNMT1) was shown to be required for restoring demethylated genomic regions induced by the methylation inhibitor 5-Aza-CdR (ref. 39). Further, a recent genome-scale study comparing DNMT occupation with DNA methylation patterns showed that a fraction of gene loci bound by DNMTs does not overlap with methylation and that the number of such loci changes dramatically on cell differentiation^1^. It has been proposed that loci bound by DNMTs, but which are not methylated, may be primed for later *de novo* methylation events that are associated with cell pluripotency and differentiation states^1^. It is possible that the bound DNMTs are inactive because of inhibition (by yet unknown mechanisms), which is removed as cell states change. *H19* expression is both temporally and spatially regulated during development, and its aberrant expression has been linked to human genetic disorders and cancer^14^,^22^. It is tempting to postulate that the enzymatic activity of DNMTs might be modulated in response to developmental cues and pathological conditions and that the *H19*–SAHH axis might be a functional link underlying these regulations.

It has become increasingly evident that the relationship between methylation and gene expression is far more complex than previously thought. Unlike the classic models linking promoter methylation to gene silencing and gene body methylation to expression, emerging models posit that the correlation between methylation and gene expression is genomic context-dependent^1^,^3^. Factors such as distance of CpGs relative to the transcription start site, their residence in CpG islands, promoters, enhancers or gene bodies as well as combinations of any of these can positively or negatively affect gene expression. Thus, it is not merely the sum or density of all methylated CpGs (which were used to label a gene as being ‘hyper' or ‘hypo' methylated) but rather the combinations of the various factors described above as well as of other yet unknown factors that determine the outcome on gene expression. Our studies reveal that *H19* regulates methylation at many genomic sites through modulating the activity of SAHH. Further investigation will certainly unravel a highly complex and context-dependent nature of this regulation. Depending on cell/tissue types, developmental stages and disease states, the biological end points of this *H19*/SAHH-mediated regulation of gene methylation are likely to be diverse. Curiously, the brain expresses very low or undetectable levels of *H19* (ref. 18) while accumulating uniquely high levels of DNA hydroxymethylation (5-hydroxymethylcytosine) during postnatal development^49^. Hydroxymethylation occurs through oxidation of the methyl group of 5-methylcytosines by TET enzymes, and this epigenetic modification is thought to be important for neuronal differentiation and function^49^,^50^,^51^. As hydroxymethylation has been implicated in the active DNA demethylation mechanism, it remains to be determined whether the activity of SAHH in the brain might be significantly lower or the activity of SAHH might be subjected to regulation by non-*H19*-mediated mechanisms. Finally, as SAM-dependent methyltransferases methylate other cellular components besides DNA, our findings open up new avenues for future investigations of other cellular components that might be regulated by the *H19*/SAHH-dependent mechanism.

## Methods

### Reagents and plasmids

Mouse monoclonal anti-SAHH (Santa Cruz Biotechnology, sc-271389), rabbit polyclonal anti-SAHH (Proteintech, 10757-2-AP), mouse monoclonal anti-HuR (Santa Cruz, sc-5261), rabbit polyclonal anti-HuR (Proteintech, 11910-1-AP), rabbit polyclonal anti-DNMT3B (Novus, NB300–516), rabbit polyclonal anti-RNAP CTD repeat YSPTSPS (phospho S5; Abcam, ab5131), rabbit polyclonal anti-β-tubulin (Abcam, ab6046), mouse monoclonal anti-β-actin (Abcam, ab8226), mouse pre-immune IgGs (Chemicon, PP54) and rabbit pre-immune IgGs (Thermo Scientific, Prod #1862244) were purchased. SiRNAs specific for mouse H19 (siH19, 4390816/n253566), SAHH (siSahh, AM16708/72248), Dnmt3b (siDnmt3b, AM16708/161533; siDnmt3b-2, 4390771/S65080) and negative siCon (AM4636) were purchased from Ambion/Life Technologies. The SAHH inhibitor DEA (sc-207632) was purchased from Santa Cruz. To construct pH19, PCR was carried out using human term placental cDNA as a template with forward 5′-aaggaaaaaagcggccgcAGCAGGGTGAGGGAGGGGGTG-3′ and reverse 5′-cgcggatccGTAACAGTGTTTATTGATGATGAGTC-3′ primers. The lower-case letters represent restriction enzyme sites for NotI and BamHI, respectively, and the upper-case letters are complementary to H19 sequence. The resulting 2.6-kb-long PCR product was inserted into pFLAG-CMV-2 (Sigma) opened with NotI and BamHI. To make pH19-S1, an annealed oligonucleotide fragment containing a 44-nt-long S1 sequence (5′-ACCGACCAGAATCATGCAAGTGCGTAAGATAGTCGCGGGCCGGG-3′) was cloned into pH19 at its 3′-end opened with BamHI. pS1 was made by inserting the S1 sequence into pFLAG-CMV-2 (Sigma) opened with NotI and BamHI.

### Cell culture and transfection

HEK293 cells (ATCC, CRL-1573) were cultured using standard protocols provided by the ATCC. Undifferentiated mouse C3H myoblasts (Sigma-Aldrich, 91031101-iVL) were maintained in growth medium (GM; DMEM, Gibco, 11965-092, supplemented with 10% fetal bovine serum, heat-inactivated, 1% penicillin/streptomycin, 1% L-glutamine and 1-mM sodium pyruvate). To prepare for differentiation, cells were seeded at a density of 1.6 × 10^5^ or 1.0 × 10^4^ cells per well in GM in 6- or 48-well plates, respectively. Differentiation was initiated 2 days later when cells became confluent by replacing GM with differentiation medium containing 2% horse serum in place of 10% fetal bovine serum. The medium was changed every other day until transfection, which was performed on days 3–4 after initiation of differentiation.

For cell transfection, mytotubes were washed once with pre-warmed OPTI-MEM immediately before addition of transfection cocktails. To prepare transfection cocktails for each well of six-well plates, 250 pmol of siCon or siH19 (with a stock solution of 5 μM) was mixed with 300 μl of OPTI-MEM by gentle pipetting. In parallel, 12.5 μl of Lipofectamine 2000 was diluted in 300 μl of OPTI-MEM by gentle pipetting. After 5-min incubation at room temperature, the two were combined by gentle pipetting. Following incubation at room temperature for 25 min, the resulting cocktail (600 μl) was directly applied to myotubes that were pre-washed with OPTI-MEM. After overnight incubation in a tissue culture incubator, the cocktail was replaced with fresh differentiation medium. Forty-eight hours later, RNA and protein were extracted for analysis.

### RNA *in situ* hybridization and SAHH immunofluorescence

Undifferentiated myoblasts were seeded in μ-Slide eight-well plates (Ibidi cat. no. 80826) at ∼2 × 10^4^ cells per well, and differentiation was initiated as described above. H19 RNA FISH experiments were carried out using the RNAscope fluorescent Multiplex system (Advanced Cell Diagnostic Inc.) according to the manufacturer's instructions. The protocol involves proteinase K treatment to remove proteins including nucleases. The system included the following components: Probe-Mm-H19-C3 (P/N: 423751-C3, L/N: 14241A; custom-designed specific for mouse H19), Blank-C1 (P/N: 300041; L/N: 14132A), 3-Plex Negative Control probe (P/N: 320371; L/N: 14105A), Pretreatment Kit-FL FF (P/N: 320842; L/N: 14099A) and Detection Kit-FL (P/N: 320851; L/N: 14098A). For SAHH immunostaining, myotubes were washed briefly with TBS (20 mM Tris-HCl, pH 7.4, 225 mM NaCl), fixed with 3% paraformaldehyde/TBS for 20 min, permeabilized with 1% SDS/TBS for 5 min and blocked with 10% BSA/0.1% goat serum/TBS for 1 h. Cells were then incubated with polyclonal anti-SAHH antibody at a dilution of 1:100 for 1 h, washed with TBS and then incubated with Alexa-488 secondary antibody (1:500 dilution, Invitrogen, Grand Island, NY) for 1 h. After washing with TBS, cells were mounted with mounting medium containing 4,6-diamidino-2-phenylindole (Vector Lab, Burlingame, CA). Images were obtained under the fluorescence microscopy (Axiovert S 100; Carl Zeiss, Oberkochen, Germany).

### RNP affinity purification and protein identification

HEK293 cells were transfected with pH19-S1, pH19 or pS1 in a 25-cm plate scale and harvested 48 h later by manual scraping and pelleted by centrifugation. After washing twice in ice-cold PBS, cell pellet was resuspended in 1.2 ml of ice-cold gentle lysis buffer (GLB) (10 mM Tris-HCl, pH 7.5; 10 mM NaCl; 10 mM EDTA; 0.5% Triton X-100; 1 mM phenylmethylsulphonyl fluoride (PMSF), 1 × proteinase inhibitor cocktail (CalBiochem, 539137); 1 mM dithiothreitol (DTT) and 1 × RNase inhibitor (Roche, 10412600)) in a 1.5-ml eppendorf tube and incubated on ice for 20 min. Cell lysate was centrifuged to remove insoluble materials. The lysate was precleared by incubation at 4 °C for 1.5 h with 40 μl of avidin agarose beads (Pierce, 20219) with NaCl added to a final concentration of 150 mM. The cleared lysate was mixed with 40 μl of streptavidin beads (packed volume, Sigma, S1638-5ML) and the tube was rotated at 4 °C for 3 h. The beads were collected and washed with 1 ml of ice-cold GLB plus (10 mM Tris-HCl, pH 7.5; 150 mM NaCl; 10 mM EDTA; 0.5% Triton X-100) five times by rotating the tube at 4 °C for 3 min each. The purified RNPs were eluted from the beads in 20 μl of 2 × SDS sample buffer by heating at 100 °C for 5 min and resolved by 10% SDS–PAGE. Following Commassie staining, the protein band at 48 kDa in the pH19-S1 lane was excised and submitted for mass spectrometry analysis by ProtTech Inc. (Norristown, PA 19403).

### RIP analysis

To prepare antibodies, 20 μl of protein A Sepharose beads were incubated with 20 μg of monoclonal anti-SAHH antibody or 20 μg of mouse pre-immune IgG in 500 μl IP buffer (0.5% Triton X-100, 200 mM NaCl, 10 mM Tris-HCl at pH 7.5 and 10 mM EDTA) at 4 °C overnight. The next day, the beads were washed three times with IP buffer and kept on ice until used. To prepare cell lysates, myotubes (from one well of a six-well plate) were harvested and cell pellets resuspended in 600 μl of freshly prepared lysis buffer (0.5% Triton X-100, 10 mM NaCl, 10 mM Tris-HCl at pH 7.5, 10 mM EDTA, 0.5 mM PMSF, 1 mM DTT, 1 × protease inhibitor cocktail (Calbiochem) and 400 units per ml RNase inhibitor). The suspensions were incubated on ice for 20 min. After removing insoluble materials by centrifugation, lysates were precleared using 10 μl of protein A sepharose (NaCl was added to a final concentration of 200 mM), followed by addition of yeast tRNA (Ambion) to a final concentration of 40 μg ml^−1^. The cleared lysates were transferred to tubes containing antibody or pre-immune IgG-coated beads, and IP was carried out by rotating the tubes at 4 °C for 3 h. Following IP, the beads were washed five times with IP buffer by adding 1 ml of the buffer and rotating the tube at 4 °C for 2 min each time. RNA was extracted from the beads using the PureLink RNA Mini Kit (Ambion, cat. no. 12183018A). Reverse transcription was performed in a 40-μl reaction volume using the Bio-Rad iScript cDNA synthesis kit, followed by quantitative PCR (qPCR).

### Genomic DNA extraction

Genomic DNA was isolated using Quick-gDNA MicroPrep (Zymo, D3021) according to the manufacturer's instructions.

### RNA extraction and RT–qPCR

Total RNAs were extracted from cells using the PureLink RNA Mini Kit (Ambion, cat. no. 12183018A). cDNA was synthesized using the Bio-Rad iSCRIPT kit (1725122) in a 20-μl reaction containing 100–500 ng of total RNA. Quantitative real-time PCR (RT–qPCR) was performed in a 15-μl reaction containing 0.5–1 μl of cDNA using iQSYBRGreen (Bio-Rad) in a Bio-Rad iCycler. PCR was performed by initial denaturation at 95 °C for 5 min, followed by 40 cycles of 30 s at 95 °C, 30 s at 60 °C and 30 s at 72 °C. Specificity was verified using melting curve analysis and agarose gel electrophoresis. The threshold cycle (*C*_t_) values of each sample were used in the post-PCR data analysis. The RT–PCR primers are listed in Supplementary Table 1.

### Western blot analysis

Cell pellets were quickly lysed in five volumes of 2 × SDS sample buffer heated at 100 °C for 5 min, with occasional vortexing. Five to ten microlitres of homogenized samples were loaded on 10% SDS gel, followed by western blot analysis. The linear dynamic range of each protein of interest was determined by serial dilutions. Bands on western blot gels were quantified using ImageJ. Uncropped blots of Fig. 1c are shown in Supplementary Fig. 7.

### QMSP analysis

Genomic DNA was extracted from myotubes in one well of six-well plates using Quick-gDNA MicroPrep. Fifteen microlitres of water were used to elute DNA from each column. For bisulfite treatment, 400–500 ng of DNA was used for each column using the EZ DNA Methylation-Gold Kit (Zymo, D5006). RT–qPCR was performed in a 15-μl reaction containing 1 μl of the eluant using iQSYBRGreen (Bio-Rad) in a Bio-Rad iCycler. The PCR primers (Supplementary Table 2) for methylated DNA were used at a final concentration of 0.3 μM in each PCR reaction. PCR was performed by initial denaturation at 95 °C for 5 min, followed by 40 cycles of 30 s at 95 °C, 30 s at 60 °C and 30 s at 72 °C. Specificity was verified using melting curve analysis and agarose gel electrophoresis. The *C*_t_ values of each sample were used in the post-PCR data analysis. Albumin DNA was used as loading controls for all QMSP normalization.

### *In vivo* SAHH activity assay

The experiments were performed in a 96-well scale using the Mouse Homocysteine (Hcy) ELISA Kit (Mybiosource, MBS260152) that allows quantitative measurement of homocysteine concentration in cell extracts, according to the manufacturer's instructions. The concentration of homocysteine in cell extracts was used as a readout for *in vivo* SAHH activity^37^, as SAHH hydrolyses SAH to homocysteine and adenosine. Briefly, myotubes were washed with cold PBS and lysed on plate in 200 μl of lysis buffer (40 mM hexadecyltrimethylammonium bromide, 75 mM Tris-HCl, pH 8.0, 1 M NaCl, 15 mM EDTA). The lysate was cleared of insoluble materials by centrifugation at 15,000*g* at 4 °C for 15 min. Immediately following the centrifugation, 100 μl of the supernatant was collected and used for SAHH activity measurement. The absorbance of the samples was determined using a FilterMax F3&F5 Multi-Mode Microplate Reader (Molecular Devices). A serial dilution of mouse Hcy was included in each assay to obtain a standard curve. The assay was performed in triplicate. The concentrations of DNA and RNA extracted from parallel wells were used as loading controls for SAHH activity normalization.

### ChIP

These experiments were performed in a six-well plate scale using the Pierce Agarose ChIP Kit (Thermo Scientific, 26156) according to the manufacturer's instructions with minor modifications. Briefly, myotubes were detached from plates in 0.25% Trypsin-EDTA and transferred to a 1.5-ml tube. Crosslinking was performed in 1% formaldehyde at room temperature for 10 min, and the reaction was stopped by glycine. ChIPs using anti-Ser-5(P)-RNAP or anti-DNMT3B antibodies (or rabbit pre-immune IgGs as a negative control) were carried out overnight at 4 °C. Levels of ChIP-purified DNA were determined with RT–qPCR (see Supplementary Table 3 for primer sequences). The relative enrichments of the indicated DNA regions were calculated using the Percent Input Method according to the manufacturer's instructions.

### Production of RNA fragments for *in vitro* analyses

Zymo Taq DNA polymerase (Zymo Research, E2001) was used to amplify hURE (128 nucleotide (nt)), mURE (122 nt) and hNSE (131 nt) using plasmid DNA containing human^29^ or mouse (accession no. NR_001592) *H19* sequences as PCR templates. All forward primers contained a T7 promoter sequence (Supplementary Table 4). The PCR products were purified using Zymo DNA clean and concentrator kit (Zymo Research, D4003) and used as templates for subsequent *in vitro* transcription. RNA fragments for EMSA and *in vitro* rSAHH activity analysis were generated by *in vitro* transcription using the T7 MEGAscript Kit (Ambion, AM1334). The resulting RNA was lithium chloride precipitated and the quality of the RNA was confirmed using electrophoresis. The RNA 3′ End Biotinylation Kit (Thermo Scientific, 20160) was used to make 3′-end biotin-labelled RNA fragments, according to the manufacturer's instructions. The biotin-labelled RNA fragments were extracted with phenol:chloroform and precipitated with 70% ethanol. Purified RNA fragments were aliquoted and stored at −80 °C until use.

### EMSA

Binding reactions were carried out in 20 μl containing 5 nM of 3′-end biotin-labelled RNA, together with indicated amounts of *Escherichia coli-*expressed, purified rSAHH, (Creative BioMart, AHCY-31342TH). Binding buffer contained 5 mM Tris-HCl, pH 7.4, 5 mM MgCl_2_, 3% (v/v) glycerol, 100 mM NaCl, 1 mM DTT, 1 × proteinase inhibitor cocktail (CalBiochem, 539137) and 1 × RNase inhibitor (Roche, 10412600)). Before mixing with the rest of the components in the binding reaction, RNA was heated at 90 °C for 3 min, followed by immediate chill on ice until use. Binding reaction was conducted at 25 °C for 30 min. At the end of incubation, 5 μl of loading buffer was added and samples loaded on 4% native polyacrylamide gels (Bio-Rad mini protein gel size), which had been pre-electrophoresed at 4 °C for 60 min in 0.5 × Tris-borate-EDTA. Electrophoresis was carried out at 4 °C at 140 V for 75 min. Following electrophoresis, components in the gels were transferred to Amersham Hybond-N+ nylon membrane (GE Healthcare, RPN303B) in 0.5 × Tris-borate-EDTA at 400 mA for 38 min at 4 °C. The membrane was then crosslinked with ultraviolet light (254 nm) for 4 min at a distance of 0.5 cm. Signals were developed using LightShift chemiluminescent EMSA kit (Thermo Scientific, 89880) according to the manufacturer's instructions.

For EMSA competition assays, rSAHH protein and labelled hURE RNA were added to a 20-μl reaction at final concentrations of 2 μM and 5 nM, respectively. Unlabelled hURE, mURE, hNSE and poly(U) (Sigma-Aldrich, P9528-10MG) were added at the indicated molar excess, followed by incubation and electrophoresis, as described above.

Band intensities of scanned films were quantified using the ImageJ software, and the data processed using the GraphPad Prism programme (Version 6.0f). The per cent bound RNA was determined as follows: [% bound complex=(bound probe−background)/(total probe−background) × 100]. All experiments contained a control reaction lacking rSAHH. *K*_d_ was calculated using nonlinear regression analysis performed with the Prism 6.0f software. *K*_d_ is the dissociation constant. Signals of complexes 1 and 2 were combined to generate probe-bound signals in *K*_d_ calculation.

### *In vitro* rSAHH activity assays

These experiments were designed to test whether binding of H19 RNA fragments to rSAHH would inhibit rSAHH enzymatic activity *in vitro*. The experiments were performed using the Adenosylhomocysteinase Activity Fluorometric Assay Kit (Biovision, K807-100) according to the manufacturer's instructions. This kit allows quantitative detection of adenosine concentration as a readout for rSAHH's ability to hydrolyse SAH *in vitro*. Briefly, 2 mg of rSAHH was pre-incubated with unlabelled RNA fragments (preheated at 90 °C for 3 min and then chilled on ice before use) in AHCY assay buffer in a total volume of 20 μl at room temperature for 20 min. At the end of incubation, an additional 30 ml of AHCY assay buffer was added, followed by addition of 50 μl of reagents containing SAH. The resulting 100 μl reaction mixture was immediately subjected to absorbance reading using a kinetic mode in a FilterMax F3&F5 Multi-Mode Microplate Reader (Molecular Devices). In the final 100-μl reaction mixture, the concentration of rSAHH was 0.4 mM, and those of the RNA fragments were 0, 0.2 and 1 nM. Relative rSAHH activities are presented with those in the absence of the RNA fragments arbitrarily set as 1.

### Methyl-MiniSeq library construction

Libraries were prepared from 200 to 500 ng of genomic DNA digested with 60 units of TaqαI and 30 units of MspI (NEB) sequentially and then extracted with Zymo Research DNA Clean & Concentrator-5 kit (Cat. no. D4003). Fragments were ligated to pre-annealed adapters containing 5′-methyl-cytosine instead of cytosine according to the Illumina's specified guidelines (www.illumina.com). Adaptor-ligated fragments of 150–250 and 250–350 bp in size were recovered from a 2.5% NuSieve 1:1 agarose gel (Zymoclean Gel DNA Recovery Kit, Zymo Research Cat. no. D4001). The fragments were then bisulfite-treated using the EZ DNA Methylation-Lightning Kit (Zymo Research, Cat. no.D5020). Preparative-scale PCR was performed and the resulting products were purified (DNA Clean & Concentrator, Zymo Research, Cat. no.D4005) for sequencing on an Illumina HiSeq.

### Methyl-MiniSeq Sequence alignments and data analysis

Sequence reads from bisulfite-treated EpiQuest libraries were identified using standard Illumina base-calling software and then analysed using a Zymo Research proprietary analysis pipeline, which is written in Python and used Bismark (http://www.bioinformatics.babraham.ac.uk/projects/bismark/) to perform the alignment. Index files were constructed using the bismark_genome_preparation command and the entire reference genome. The non_directional parameter was applied while running Bismark. All other parameters were set to default. Filled-in nucleotides were trimmed off when doing methylation calling. The methylation level of each sampled cytosine was estimated as the number of reads reporting a C, divided by the total number of reads reporting a C or T. Fisher's exact test or *t*-test was performed for each CpG site, which has at least five reads coverage, and promoter, gene body and CpG island annotations were added for each CpG included in the comparison.

### RNA-seq and data analysis

Mouse C3H myoblasts were maintained in six-well plates. Myotubes were transfected with siCon or siH19 in triplicates for 40 h following initiation of differentiation. Cells were harvested for RNA extraction 48 h post transfection using the Purelink RNA mini kit (Cat. no. 12183018A). RNA-seq libraries were prepared using the Illumina TruSeq Stranded Total RNA LT kit with Ribo-Zero Human/Mouse/Rat, setA (Cat. no. rs-122–2201) according to the sample preparation protocol. Briefly, 1 μg total RNA was subjected to Ribo-Zero depletion to remove rRNAs. The remaining RNA was purified, fragmented and primed with random hexamers for cDNA synthesis. After first and second cDNA synthesis, cDNA fragments were adenylated and then ligated to indexing adapters. The cDNA fragments were enriched by PCR, purified and then sequenced on an Illumina NextSeq500 using paired-end chemistry and 76-bp cycles. Sequences are available from the GEO with accession number GSE73014.

Illumina BaseSpace (https://basespace.illumina.com/)-embedding tools were used to analyse the RNA-seq data. TopHat Alignment 1.0.0 app was used to map sequencing reads to mm10 genome. Cufflinks Assembly & DE 1.0.0 app containing Cufflinks 2.1.1 and Cuffdiff 2.1.1 was applied to assemble mapped transcripts and calculate differential expression of genes and transcripts.

### Statistical analysis

All data (unless otherwise indicated) are presented as mean±s.d. Statistical analyses were performed using the Statistical Package for the Social Science computer software version 17.0 (IBM SPSS Statistics, Chicago, IL, USA). The Student's *t*-test was used to compare differences between quantitative variables. The Fisher's exact text was used when appropriate, for comparing categorical variables (contingency tables). *P* values at 0.05 or smaller were considered significant.

## Additional information

**Accession codes:** RNA-seq and methylation profile data are available at the GEO under accession code: GSE73014.

**How to cite this article:** Zhou, J. *et al.* H19 lncRNA alters DNA methylation genome-wide by regulating S-adenosylhomocysteine hydrolase. *Nat. Commun.* 6:10221 doi: 10.1038/ncomms10221 (2015).

## Supplementary Material

Supplementary Information Supplementary Figures 1-7 and Supplementary Tables 1-4

Supplementary Data 1 Top 2000 genes strongly hyper- or hypo-methylated in the siH19 relative to siCon control cells. Strongly hypermethylated: 33100% more methylated in siH19 than siCon (pvalue < 0.05); Strongly hypomethylated: 33100% less methylated in siH19 than siCon (pvalue < 0.05). siH19 meth_ratio/siCon meth_ratio: these ratios compare the number of methylated C's to the total number of reads containing that specific CpG. Meth diff: the difference was calculated by subtracting the siCon sample from the siH19 sample.

Supplementary Data 2 Genes at least 1.5-fold differentially expressed in siH19 over siCon cells. With 1.5-fold change cut-off, 1131 genes or transcripts were found significantly upregulated in siH19 relative to siCon cells, meanwhile 508 genes or transcripts were significantly downregulated. Significant value “TRUE” means that the p value calculated from either siCon or siH19 triplicate samples is < 0.05.
