# Supplementary material for: H19 lncRNA alters DNA methylation genome wide by regulating S-adenosylhomocysteine hydrolase
Source: Nat Commun. 2015 Dec 21;6:10221. doi: 10.1038/ncomms10221 (PMC4703905; doi:10.1038/ncomms10221)
Supplement: Supplementary Information — Supplementary Figures 1-7 and Supplementary Tables 1-4 [file ncomms10221-s1.pdf]

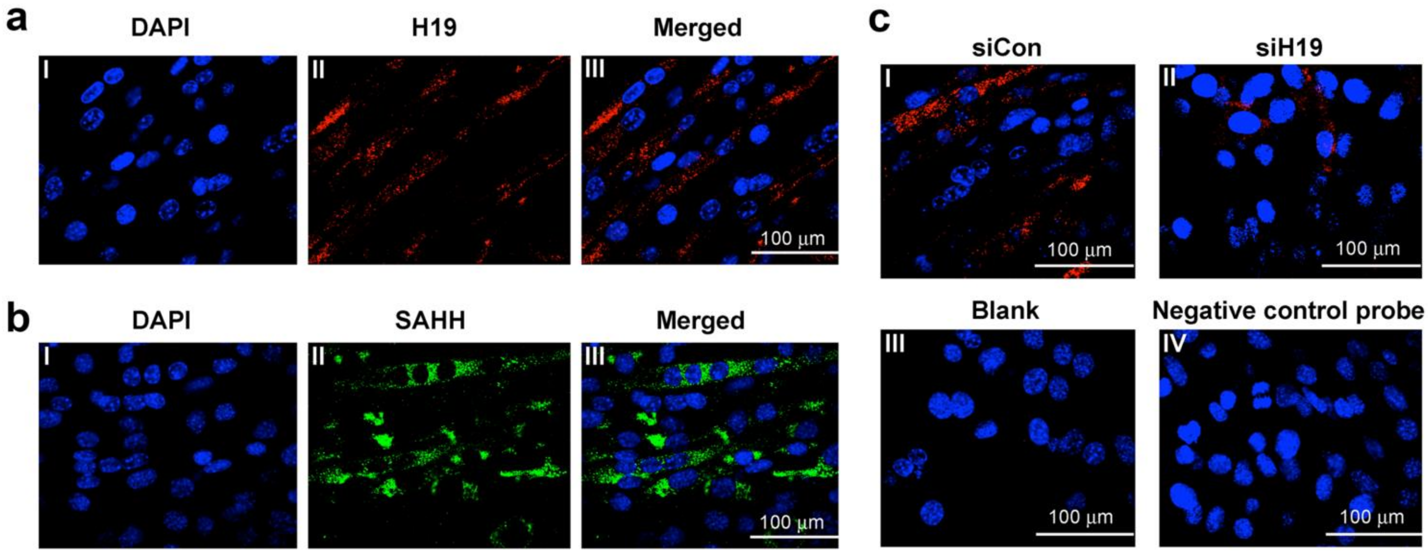

Supplementary Figure 1. Subcellular localization of H19 and SAHH. (a) RNA fluorescence *in situ* hybridization (RNA FISH) to visualize endogenous H19 in C3H mytotubes. Panel I: DAPI staining showing cell nuclei in blue; Panel II: FISH showing H19 signals in red; Panel III: superimposed image of I and II showing predominantly cytoplasmic H19. **(b)** Immunofluorescence (IF) to visualize endogenous SAHH in C3H mytotubes. Panel I: DAPI staining showing cell nuclei in blue; Panel II: IF showing SAHH protein signals in green; Panel III: superimposed image of I and II showing predominantly cytoplasmic SAHH. **(c)** Quality control RNA FISH confirming the specificity of the H19 probe. Myotubes were transfected with siCon (panel I), siH19 (panel II), or mock transfected (panes III and IV). RNA FISH were performed 48 h later. In I and II, the H19-specific probe was applied; in III and IV, no probe and a negative control probe were applied, respectively.

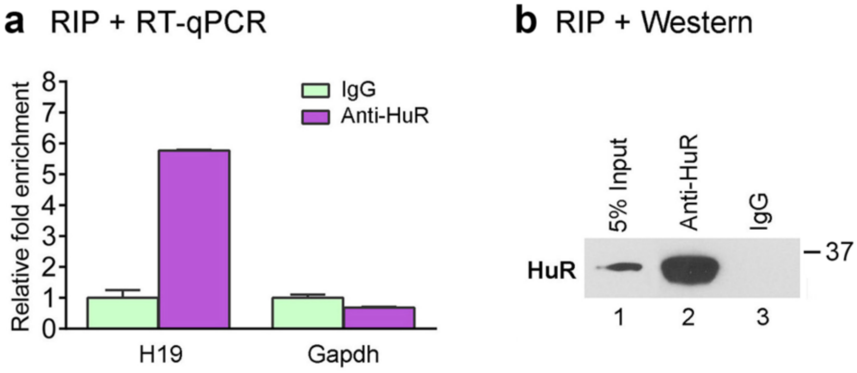

Supplementary Figure 2. H19 is enriched in HuR-containing RNPs. (a) RIP with rabbit polyclonal anti-HuR or preimmune rabbit IgGs from myotube extracts. RNA levels in immunoprecipitates were determined by RT-qPCR. Levels of H19 and Gapdh mRNA are presented as fold enrichment in anti-HuR relative to IgG immunoprecipitates. Numbers are mean ± SD (n=3). (**b**) Immunoprecipitation using polyclonal anti-HuR (lane 2) or IgG (lane 3), followed by Western blot analysis using a mouse monoclonal anti-HuR. Five percent input was loaded in lane 1. The HuR band is marked. Molecular size in kDa is marked on the right.

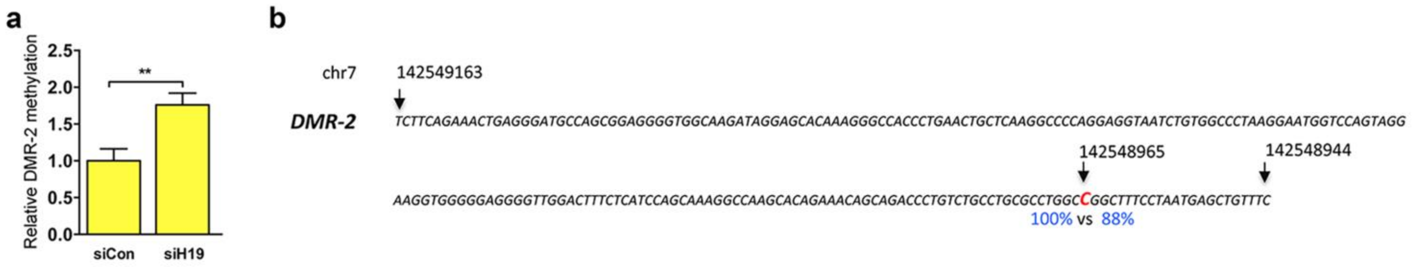

Supplementary Figure 3. CME methylation at DMR-2. (**a**) Relative DMR-2 methylation assessed using QMSP. Methylation in the siCon cells was arbitrarily set as 1. Numbers are mean ± SD (n=3). **, p < 0.01. (**b**) Sequence of the DMR-2 amplified using MSP primers, with the differentially methylated cytosine highlighted in red. Numbers above the sequence mark positions of the indicated nucleotides in the chromosome. Numbers below (in blue) indicate percentage of methylation in the siH19 (left) versus siCon (right) cells as determined by genome-wide methylation mapping.

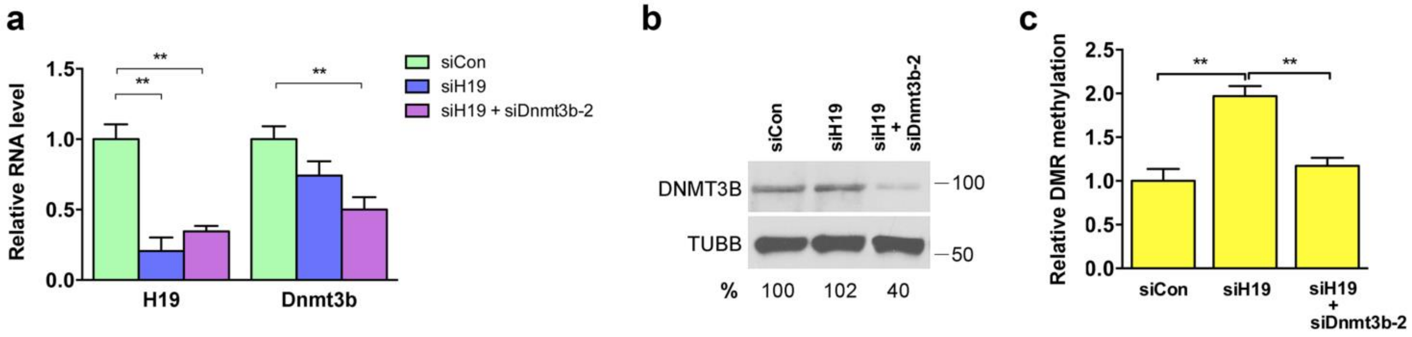

Supplementary Figure 4. Effects of siDnmt3b-2 on DMR methylation. (**a**) Myotubes were transfected with siCon, siH19, or siH19 plus siDnmt3b-2, followed by RNA extraction and RT-qPCR analysis 48 h later. Relative RNA levels of H19 and Dnmt3b are presented. (**b**) Western blot results representative of three independent transfection experiments (performed as described in **a**) are shown. Antibodies specific for DNMT3B and beta-tubulin were used on the top and bottom blots, respectively. Numbers underneath the blots indicate DNMT3B protein levels after normalization against TUBB loading controls with that in the siCon cells arbitrarily set as 100%. (**c**) Myotubes were transfected with siCon, siH19, or siH19 plus siDnmt3b-2. DMR methylation was assessed 48 h post-transfection using MSP. Numbers are mean ± SD (n=3). **, p < 0.01.

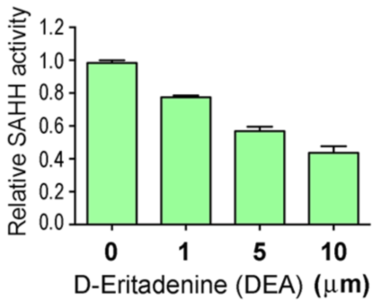

Supplementary Figure 5. Dose-dependent inhibition of SAHH activity by DEA. Myotubes were incubated without or with the SAHH inhibitor DEA at a final concentration of 1, 5, or 10 uM. Cell lysate was prepared 24 h later and SAHH enzymatic activity was measured using the SAHH kit according to the manufacturer's instruction. Relative SAHH activities are presented with the no DEA treatment arbitrarily set as 1.

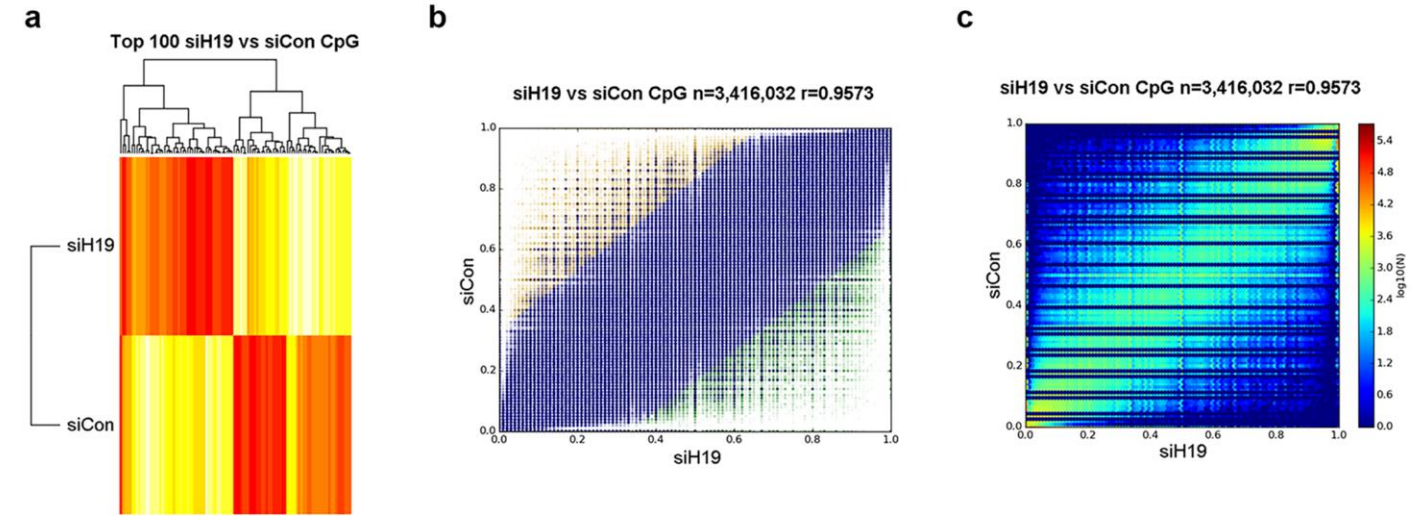

Supplementary Figure 6 (a) Hierarchical Clustering. Samples were grouped based on similarity for the top 100 differentially methylated CpG sites covered in the assay. Yellow represents high levels of DNA methylation, and red represents low levels of DNA methylation. (**b**) Pairwise Scatter Plot. Scatter plots depict correlation between two samples. For replicates, one would expect to see a straight "line" with slope=1, demonstrating perfect 1:1 correlation. Blue dots show sites with statistically insignificant differences between samples, significant difference sites are colored yellow and green. (**c**) Scatter Plot similar to **b** but using color to indicate the density of the CpG sites.

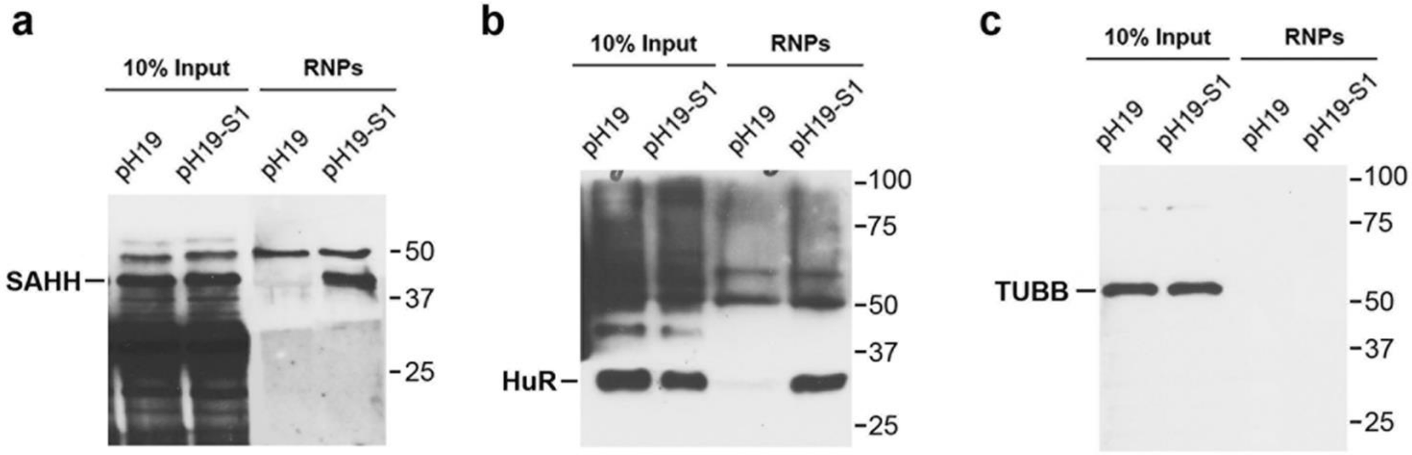

Supplementary Figure 7. SAHH and HuR are specifically enriched in H19-containing RNPs. pH19 or pH19-S1 were transfected into HEK293 cells, followed by RNP affinity purification and Western blot analysis using rabbit polyclonal antibodies specific for SAHH (**a**), HuR (**b**), or beta-tubulin (TUBB) (**c**). Positions of SAHH, HuR and TUBB are marked on the left, with protein size markers in kDa shown on the right.

Supplementary Table 1. Real-time PCR primer sequences

Gene	Forward primer	Reverse primer
Nctc1	5'-CAACTCCTACCACCAAAGCA-3'	5'-TCCATCTCCCTTGCTGTATC-3'
H19	5'-CCTCAAGATGAAAGAAATGGTGCTA-3'	5'-TCAGAACGAGACGGACTTAAAGAA-3'
Gapdh	5'-CCTTCATTGACCTCAACTACAT-3'	5'-CAAAGTTGTCATGGATGACC-3'
Sahh	5'-ATCCTTGGCCGGCACTTT-3'	5'-TTCTTTAGCCAGTAGCGGTCCA-3'
Dnmt3b	5'-TTCAGTGACCAGTCCTCAGACACGAA-3'	5'-TCAGAAGGCTGGAGACCTCCCTCTT-3'
Beta-tubulin	5'-CGTGTTCGGCCAGAGTGGTGC-3'	5'-GGGTGAGGGCATGACGCTGAA-3'

Supplementary Table 2. QMSP primer sequences

Gene	Forward primer	Reverse primer
Nctc1-MDR	5'-GCGGGTTTTTCGTTATTTCG-3'	5'-ACAAAAATATTCTTTATCCGAAAATATCG-3'
Nctc1-MDR-2	5'-TTTTTAGAAATTGAGGGATGTTAGC-3'	5'-AAAACAACTCATTAAAAAAACCGAC-3'
Bmp8b	5'-GGAAGGTATTTAGTAGATAGGAGCG-3'	5'-ATTAAACTAACCGTCGAAACGAA-3'
Casp2	5'-GGTGGTGCGTGTGTAGTTC-3'	5'-ACCTACTCCTCCTATCAACCG-3'
Cdt1	5'-TTTGTTTTTAATTTTTTAGTTTCGG-3'	5'-TAAACACCAAAAAACGCATACG-3'
Foxa2	5'-GGGTTTTGAATAAATTTTTAGAAATTAC-3'	5'-AAAACATTCTAACAACCCGAA-3'
Setd2	5'-GTAGGTTAGGTTTGGTTTGGTC-3'	5'-CAACCCCTTAAAAAAACACG-3'
Albumin	5'-GTGAGAATTGTAGAGCAGTGCTGTC -3'	5'-ACATTGCTCAGCACAGATCCAC-3'

Supplementary Table 3. ChIP PCR primer sequences

Gene	Forward primer	Reverse primer
Nctc1-Exon 1	5'-ACATTCAGGCAGTGACCAAT-3'	5'-GCTCCGACCTGAATATCTTG-3'
H19-Exon 1	5'-CCAAAAGTAACCGGGATGAA-3'	5'-CTGGAGTCTGGCAGGAATGT-3'
Neg	5'-GGTCCGATCTAAGCCCTAGCATT-3'	5'-TTCCTGGCACTAGCCAGTCTCTTT-3'
Bmp8b-DMR	5'-ATAGCTTGGCGCACAGAACC-3'	5'-CGAGGGTGGGGCACATTC-3'
Casp2-DMR	5'-GGCCCAGCGTTTGTTTGTTT-3'	5'-TTCCTGTGCAGGGAGGACT-3'
ChNctc1-DMR	5'-TCTCATCCAGCAAAGGCCAA-3'	5'-AAAACCCCAGATCAAAGGGCT-3'
FoxA2-DMR	5'-AGCTTGAAGGTCTGGTGCC-3'	5'-CGTAGACTTCCGAGCGAGC-3'

Supplementary Table 4. T7 PCR primer sequences

Gene	Forward primer	Reverse primer
Human H19 U-rich element 128nt	5'-TAATACGACTCACTATAGGGCTTCTTTTTCATCCTT-3'	5'-CAAATGACTTAGTGCAAATTAAATTCAGAAGGGAC-3'
Human H19 G-rich element 131nt	5'-TAATACGACTCACTATAGGGCTGGTGCGGAGAGGGC-3'	5'-TCGCCTAGTCTGGTCTCGCCCCATGCC-3'
Mouse H19 U-rich element 122nt	5'-TAATACGACTCACTATAGGGCCTGTCTTTTTCTTCT-3'	5'-TGCAAATTCAAAAGGGGGTAAATGGG-3'

TAATACGACTCACTATAGGG is T7 promoter.
